# Depressive symptomatology and personality traits in patients with symptomatic and asymptomatic peripheral arterial disease

**DOI:** 10.1186/s12872-020-01586-y

**Published:** 2020-06-22

**Authors:** Gergely Tóth-Vajna, Zsombor Tóth-Vajna, Piroska Balog, Barna Konkolÿ Thege

**Affiliations:** 1 Institute of Behavioral Sciences, Semmelweis University, Nagyvárad tér 4. XX. emelet, Budapest, 1089 Hungary; 2 Heart and Vascular Center, Department of Vascular Surgery, Semmelweis University, Városmajor utca 68, Budapest, 1122 Hungary; 3 Waypoint Research Institute, Waypoint Centre for Mental Health Care, 500 Church St, Penetanguishene, Ontario Canada; 4 Department of Psychiatry, University of Toronto, 250 College St, Toronto, Ontario Canada

**Keywords:** Peripheral arterial disease, Depression, Neuroticism, Big five personality traits

## Abstract

**Background:**

The aim of this study was to examine the relationship of depressive symptomatology and personality traits with peripheral arterial disease (PAD).

**Methods:**

The sample of this cross-sectional study comprised of 300 individuals (*M*_*age*_ = 65.3 ± 8.7 years, 61.0% female) recruited from the offices of 33 general practitioners. Based on at-rest ankle-brachial index (ABI) values and claudication symptoms, four subsamples were formed: clear PAD-positive, clear PAD-negative, ABI-negative but symptomatic, and a non-compressible-artery group. The concurrent role of depression (assessed by a shortened version of the Beck Depression Inventory) and personality traits (measured by the Big Five Inventory) in predicting PAD status was examined using multinomial logistic regression – controlled for sex, age, hypertonia, diabetes, smoking, hazardous drinking, and body mass index.

**Results:**

Depressive symptomatology was significant in predicting peripheral arterial disease status even after controlling for both traditional risk factors and personality traits. Among the Big Five personality traits, neuroticism showed a significant, positive relationship with PAD – independently of depression.

**Conclusions:**

Patients with PAD – even those with asymptomatic forms of the disease – are at higher risk for suffering from depression compared to individuals without PAD, independently of neuroticism, other Big Five personality dimensions or traditional risk factors for cardiovascular diseases.

## Background

As a result of population growth, global ageing and diabetes, the number of patients with peripheral arterial disease (PAD) has increased by 23% in the last decade [1]. The largest national epidemiological study estimated the prevalence of PAD to be 14.4% in Hungary among individuals suffering from hypertension [2]. There is no population-based prevalence data from Hungary in this regard, but the number of major amputations is estimated to be the triple of the international average, suggesting a higher prevalence of PAD in the general Hungarian population compared to other countries [3, 4]. Traditional risk factors of PAD include hypertension, smoking and hyperlipidemia but there is an increasing attention on the psychological aspects and their role in the pathomechanism of PAD. Psychosocial factors might also influence the way in which the symptoms of PAD are reported, coped with, and treated [5]. Depression is the most frequently assessed psychosocial factor among patients with PAD. According to these investigations, there is a correlation between PAD and depression [6] and the prevalence of depression among patients with PAD is comparable to the prevalence estimated in CVD patients (cross-sectional estimates range from 11 to 48%) [7].

The depression-PAD association is hypothesized to be bidirectional, influenced by several factors. On the one hand, patients with depressive symptoms are more likely to experience later PAD symptoms [8], while on the other hand, individuals with PAD are at higher risk for developing depression [9]. Moreover, among individuals with PAD, depression is associated with higher mortality rates compared to the mortality risk in those without depression, even though adjusting for baseline PAD severity (baseline 6-min walk performance) attenuates this association [9].

The PAD-depression association may also be influenced by personality factors. For instance, Type D personality independently predicted individual differences in impaired quality of life and depressive symptoms in patients with PAD [10]. To date, extant studies on personality among patients with PAD have focused on individual, isolated personality traits or specific personality patterns: Type-D personality [10], Type A Behavior Pattern [11] or hostile personality [12]. However, no studies have been devoted to a better understanding of the relationships between PAD and a wider range of personality traits simultaneously, which led to the authors of a related systematic review to explicitly call for the development of such a research agenda [5] including Big Five personality traits [13].

Further, even though it is well documented that depression is the main modifiable psychological risk factor in PAD [6] and that personality factors not only correlate with depression [14] but might play an important role in the incidence of depressive symptoms [15–17]; very few studies analyzed personality traits and depressive symptoms within the same study [12], let alone in the same statistical model [10] when predicting PAD status. Importantly, Big Five personality traits, for instance lower openness and extraversion, have been linked to poorer cardiovascular outcomes [18] and neuroticism specifically was also linked to a higher risk of coronary heart disease [19] but these personality traits have never been studied in terms of their associations with PAD.

To sum up, studies to date aiming to investigate depression and personality traits simultaneously among PAD patients have not considered a wide enough range of personality traits. Moreover, none of the previous studies has considered the multifaceted clinical presentation of PAD either. Therefore, the aim of the present study was to investigate the relationship between Big Five personality traits, depression, and PAD – controlling for well-known risk factors for cardiovascular diseases. When doing so, three different presentations of PAD were considered to provide a more nuanced picture of the relationship between the psychological variables and disease progress. We hypothesized, that both symptomatic and asymptomatic patients with PAD would show higher level of depressive symptomatology than those who do not show either objective symptoms or report subjective complaints related to peripheral arterial disease. We also hypothesized that both symptomatic and asymptomatic patients with PAD would show higher level of neuroticism.

## Methods

### Participants and procedure

The protocol of the current study was approved by the Scientific and Research Ethics Committee of the Medical Research Council, Semmelweis University (ETT TUKEB 285/2015) and was carried out in accordance with the tenets of the Declaration of Helsinki. Data collection took place between November 2015 and February 2018, with participants recruited from the practices of 33 general practitioners from across Hungary.

PAD usually appears after the age of 50, with an exponential increase after the age of 65 years. According to governmental regulations of the country of study, it is obligatory to record every patients’ ankle-brachial index (ABI) values every two years after reaching the age of 45; therefore, the target population included men and women aged 45 or older with at least one major vascular risk factor (current smoking, type 2 diabetes, or hypertension). The current sample is a subsample of a larger epidemiological study, which involved 816 individuals and investigated the prevalence of PAD in the primary health care setting in Hungary [3]. Out of this larger pool of participants, 300 (*M*_*age*_ = 65.3 years, *SD* = 8.7 years; 61.0% female) individuals were approached and agreed to participate in the present study, that is, agreed to complete the psychological test battery on top of the medical screening. This way the sample should be considered as a convenience one with a response rate of 100%, which probably is the consequence of the primary care environment (family physician’s office) and the generally high compliance with medical staff (assessors were resident physicians) in these settings in post-socialist countries. All participants provided written informed consent.

Participants’ medical history and the presence of major cardiovascular risk factors were recorded based on the health records kept by their general practitioners. The in-person examination started by completing the Edinburgh Claudication Questionnaire, a validated and frequently used method of screening for intermittent claudication. The questionnaire has a sensitivity of 80–90% and a specificity of over 95% [20]. Second, the basic body measurements (height and weight) were performed. After a 5-min rest, blood pressure and pulse were measured on both upper extremities (using a blood pressure manometer, Bosch Konstante) three times. Following current recommendations for the calculation of the ankle-brachial index (ABI) [1], systolic pressure on all four extremities was also measured with a continuous wave Doppler-US instrument at 8 MHz (multiDOPPY). A more detailed description of the assessment methodology can be found in a former study [3].

Taking into consideration that individuals in the early stages of PAD either do not experience or under-report claudication symptoms (lower extremity pain) and thus PAD frequently remains undetected and untreated [21], we decided to consider a more complex taxonomy than the simple presence or absence of PAD – also suggested by current clinical guidelines [1]. Accordingly, based on at-rest ABI values and symptoms, four patient-groups were determined. Patients with negative at-rest ABI results without any symptoms indicating sclerosis were considered as ‘clear PAD-negative’. Patients with normal at-rest ABI values but whose Edinburgh Questionnaire results revealed symptoms of intermittent claudication (e.g., pain following walking uphill or climbing stairs) were coded as ‘ABI-negative-symptomatic’. The ‘clear PAD-positive’ group comprised of patients whose ABI results were positive and clearly suffered from atherosclerosis, asymptomatic or symptomatic stenosis or occlusion reducing the blood flow. Patients, whose major arteries are hardened for various reasons (such as medial sclerosis), have non-compressible arteries. Due to this, blood pressure values at the ankle often show false high values; the Doppler Index is over 1.4. This subgroup of participants was labelled as the ‘non-compressible-artery group’ [3].

### Psychological instruments

Depressive symptoms were measured with the shortened Hungarian version of the Beck Depression Inventory (BDI), which is a 9-item questionnaire to assess depression severity [22]. Each item is scored on a 4-point scale ranging from 0 (not at all characteristic of me) to 3 (very characteristic of me). Internal consistency of the scale proved to be excellent in the current sample (Cronbach’s *α* = .86). To allow international comparability, the total score of the 9-item version was transformed to its equivalent in the 21-item original version by multiplying the total score by 2.22. The cut off score indicating the presence of at least mild depression was therefore identical to that in the international literature (≥10).

Personality dimensions (extraversion, agreeableness, conscientiousness, neuroticism and openness) were measured with the Big Five Inventory (BFI-44) [23]. On the 44-item questionnaire, each item was rated on a 5-point scale ranging from 1 (strongly disagree) to 5 (strongly agree). Internal consistency of the dimensions was acceptable, good or excellent in the current sample (Cronbach’s *α*-s of .89, .75, .71, .84*,* and .90, respectively).

Hazardous drinking was measured with the Alcohol Consumption Questions of the AUDIT (AUDIT-C) [24]. This is a 3-item tool for screening hazardous drinking habits. Each question is scored from 0 to 4. Four points or above in men, 3 points or above in women are indicative of hazardous drinking. The advantage of the shortened version is its easy use in primary care. Internal consistency in the current sample was excellent (Cronbach’s *α* = .84).

### Statistical analyses

The Kolmogorov-Smirnov test indicated that the distribution of all continuous variables differed significantly from the normal distribution. Therefore, when investigating the relationship between peripheral arterial disease status and these variables, the non-parametric Kruskal-Wallis test was used (eta squared was used to express effect size based on the formula suggested by Tomczak & Tomczak [25]). When examining the association between the dependent variable and the categorical independent variables, the chi square test was employed (Cramer’s V was reported to express effect size).

On the multivariate level, a multinomial logistic regression analysis was conducted to investigate the role of all independent variables in differentiating between those intact from peripheral arterial disease (‘clear PAD-negative’) versus those affected (‘ABI-negative-symptomatic’, ‘non-compressible-artery’ and ‘clear PAD-positive’). All of the above analyses were carried out using the Statistical Package for the Social Sciences, Version 25 (IBM SPSS, 2017). To estimate the risk of Type II error, post hoc power analysis was carried out using the G*power 3.1.9.2 software. Achieved power was calculated considering the type of the multivariate analyses (z test for logistic regression), alpha level (0.05), sample size (*N* = 165–212, depending on the subsamples compared), effect size (odds ratio for the given independent variable), and explained variance from the full regression model.

## Results

The descriptive data indicated that the prevalence of depression was 63% in the clear PAD-positive subgroup, 59% in those who were symptomatic, but whose ABI values did not show abnormalities, and only 20% in the non-compressible-artery group in contrast to the 8% prevalence rate among those without any signs of PAD. The results indicated that the relationship between depression and PAD is strong (Table 1). Results of the further bivariate analyses indicated that all independent variables but hypertonia and diabetes status were significantly associated with peripheral arterial disease status. In the case of sex, smoking, and neuroticism, the effect size was large, and in the case of continuous depressive symptom scores the effect size was very large (Table 1). The data showed that those without any signs of peripheral arterial disease reported lower levels of depressive symptomatology than the rest of the sample.

**Table 1:** Characteristics of the sample stratified by peripheral arterial disease status

	Clear-PAD-negative (*n* = 126)	ABI-negative-symptomatic (*n* = 49)	Non-compressible-artery group (*n* = 39)	Clear-PAD-positive (*n* = 86)	Comparison of the groups
Sex					*χ*^*2*^ = 9.047, *p* = .029, V = .174
Female	87 (69.05)	27 (55.10)	26 (66.67)	43 (50.00)	
Male	39 (30.95)	22 (44.90)	13 (33.33)	43 (50.00)	
Age (years)	63.52 (8.88)	66.14 (7.30)	66.62 (9.12)	66.90 (8.45)	*K-W χ*^*2*^ = 10.955, *p* = .012, η^2^ = .027
Hypertonia					*χ*^*2*^ = 3.530, *p* = .317, V = .108
No	31 (24.60)	8 (16.33)	9 (23.08)	13 (15.12)	
Yes	95 (75.40)	41 (83.67)	30 (76.92)	73 (84.88)	
Diabetes					*χ*^*2*^ = 3.210, *p* = .360, V = .103
No	83 (65.87)	33 (67.35)	20 (51.28)	53 (61.63)	
Yes	43 (34.13)	16 (32.65)	19 (48.72)	33 (38.37)	
Smoking					*χ*^*2*^ = 17.295, *p* = .001, V = .240
No	108 (85.71)	34 (69.39)	31 (79.49)	53 (61.63)	
Yes	18 (14.29)	15 (30.61)	8 (20.51)	33 (38.37)	
Hazardous drinking	1.11 (1.33)	1.06 (1.65)	1.23 (1.66)	2.05 (2.30)	*K-W χ*^*2*^ = 11.506, *p* = .009, η^2^ = .028
Body mass index	30.91 (5.49)	29.48 (5.28)	31.85 (6.33)	28.19 (4.59)	*K-W χ*^*2*^ = 16.489, *p* = .001, η^2^ = .046
Extraversion	29.44 (6.67)	26.02 (7.15)	29.97 (7.05)	23.13 (6.87)	*K-W χ*^*2*^ = 45.823, *p* < .001, η^2^ = .145
Agreeableness	35.75 (4.26)	34.86 (4.87)	36.32 (5.06)	31.45 (5.70)	*K-W χ*^*2*^ = 33.824, *p* < .001, η^2^ = .104
Conscientiousness	37.58 (4.68)	36.61 (4.07)	37.67 (4.35)	35.10 (4.38)	*K-W χ*^*2*^ = 16.470, *p* = .001, η^2^ = .046
Neuroticism	21.85 (5.10)	26.37 (5.47)	24.05 (6.17)	28.58 (6.14)	*K-W χ*^*2*^ = 63.708, *p* < .001, η^2^ = .205
Openness	32.32 (8.57)	28.02 (8.22)	33.31 (9.25)	25.66 (6.16)	*K-W χ*^*2*^ = 41.448, *p* < .001, η^2^ = .130
Depression (continuous)	3.82 (6.29)	12.28 (8.41)	6.03 (6.15)	16.31 (10.42)	*K-W χ*^*2*^ = 98.393, *p* < .001, η^2^ = .322
Depression (categorical)	10 (7.94)	29 (59.18)	8 (20.51)	54 (62.79)	*χ*^*2*^ = 87.325, *p* < .001, V = .540

Note. Descriptive values are frequencies (and percentages) for categorical variables, while means (and standard deviations) for continuous variables. V: Cramer’s V; K-W *χ*^*2*^: Kruskal-Wallis *χ*^*2*^

Similar findings emerged from the multivariate analyses (Table 2): results of the multinomial logistic regression analysis (*χ*^*2*^ = 181.5, *p* < .001, Cox and Snell *R*^*2*^ = .456) indicated that depressive symptomatology was significant in predicting peripheral arterial disease status even after controlling for both traditional risk factors and personality traits. In each pair of comparison, increased level of depressive symptomatology was associated with a higher likelihood (OR: 1.22–1.45) of presenting with symptoms of peripheral arterial disease. Among the Big Five personality traits, neuroticism showed a consistent, positive relationship with peripheral arterial disease: in each comparison, those with a higher level of neuroticism had a higher likelihood (OR: 1.10–1.12) of suffering from the symptoms of peripheral arterial disease. In most cases (with the single exception of Agreeableness in case of the ABI-negative-symptomatic subgroup), the other personality traits were not statistically significant in predicting peripheral arterial disease status; however, the achieved power was very low (.05–.14) (Table 2).

**Table 2:** Predictors of peripheral arterial disease status (multinomial logistic regression)

	ABI-negative-symptomatic†				Non-compressible-artery group †				Clear-PAD-positive†			
	*OR*	*95% CI*	*p*	*power*	*OR*	*95% CI*	*p*	*power*	*OR*	*95% CI*	*p*	*power*
Sex (male)	1.888	0.798–4.466	.148	.771	1.145	0.453–2.892	.775	.128	1.654	0.723–3.786	.234	.676
Age	1.046	0.994–1.101	.082	.071	1.059	1.005–1.116	.032	.076	1.057	1.009–1.107	.020	.079
Hypertonia	1.742	0.612–4.958	.299	.672	1.059	0.393–2.858	.909	.076	1.944	0.719–5.257	.190	.869
Diabetes	1.180	0.508–2.742	.700	.157	1.723	0.742–3.998	.205	.634	1.549	0.712–3.372	.270	.574
Smoking	5.757	2.089–15.868	.001	1.00	3.515	1.183–10.448	.024	.998	6.829	2.589–18.018	< .001	1.00
Hazardous drinking	0.925	0.702–1.220	.582	.090	1.100	0.831–1.456	.505	.099	1.211	0.967–1.516	.095	.202
Body mass index	0.987	0.914–1.067	.746	.055	1.056	0.981–1.137	.146	.075	0.937	.868–1.012	.099	.086
Extraversion	1.031	0.945–1.125	.492	.063	1.033	0.938–1.138	.505	.064	1.005	0.926–1.090	.914	.052
Agreeableness	1.160	1.039–1.294	.008	.142	1.105	0.985–1.240	.089	.102	1.042	0.943–1.151	.420	.071
Conscientiousness	1.078	0.978–1.189	.129	.088	1.051	0.950–1.162	.335	.073	1.040	0.950–1.138	.399	.070
Neuroticism	1.097	1.004–1.198	.041	.099	1.120	1.025–1.225	.012	.111	1.102	1.015–1.196	.021	.109
Openness	0.973	0.901–1.051	.486	.062	1.032	0.953–1.118	.437	.063	1.012	0.941–1.088	.750	.056
Depression(continuous)	1.451	1.241–1.697	< .001	.409	1.218	1.024–1.448	.026	.181	1.436	1.240–1.662	< .001	.450

†Reference category: clear-PAD-negative

*OR*: odds ratio; *CI*: confidence interval for the odds ratio; *power*: achieved (post hoc) power

## Discussion

The aim of the present study was to shed light on the complex relationships existing between personality traits, depression, and peripheral arterial disease, taking PAD severity into consideration as well. The results showed that patients with PAD – even those suffering from asymptomatic forms of the disease – are at higher risk for suffering from depression compared to people without PAD, independently of neuroticism or other Big Five personality traits.

On the one hand, our findings are consistent with prior studies indicating a larger prevalence of depression in individuals with PAD than in those without [7]. On the other hand, our results indicate a higher prevalence of depression in the ‘clear PAD positive’ subgroup of our patients (63%) than what was found in studies included in Sliwka’s systematic review, where the rate of PAD-symptomatic patients presenting with depressive symptoms varied between 9.4 and 53% [5]. This difference in depressive symptom prevalence might be partially related to the different assessment approaches to depression in previous studies [5], but most likely it is not independent of the elevated level of depression in the Hungarian general population [26] either. It is worth mentioning that very few previous studies have considered asymptomatic PAD patients, who also reported higher rate of depressive symptoms in the present study (compared to the ‘clear PAD negative’ subgroup) similar to patients with atypical leg symptoms in the study of Smolderen and colleagues [27] who reported more impaired mood than their asymptomatic counterparts.

In line with our second hypothesis, a higher level of neuroticism has also been associated with an increased likelihood of PAD. Neuroticism – the tendency to more frequently experience negative emotions such as anger, sadness, anxiety, worry, low self-consciousness, hostility, irritability and vulnerability – showed a consistent positive relationship with peripheral arterial disease: in each comparison, those with a higher level of neuroticism had a higher likelihood of suffering from the symptoms of peripheral arterial disease, independently from depressive symptomatology. In previous studies, neuroticism was found to be connected with negative life events in a bidirectional way [28] as was linked to CVD risk factors such as smoking [29], physical inactivity [30], higher body mass index [31] and an increased prevalence of metabolic syndrome [32]. High neuroticism also proved to be a risk factor for coronary heart disease- but not stroke mortality [19, 33].

It is well known that neuroticism is a significant predictor of the incidence of depressive symptoms [15–17] and it has been linked to late-life depression [34] but very few studies analyzed personality traits and depressive symptoms simultaneously in predicting PAD status [10, 12]. A recent longitudinal study, taking into consideration the complex relationship between neuroticism, depression and CVD, found that both neuroticism and depression were associated with increased risk of CVD; moreover, a synergistic interaction between neuroticism and depression status on further risk of CVD was found [35]. However, an unexpected conclusion has been drawn in a population-based cohort study with a 9-year follow-up: depression was predictive for future stroke in the context of low neuroticism only [36] [later, this study has been criticized for its methodological shortcomings such as high prevalence of missing data, poor study design, and the low number of stroke participants [35]]. The authors of this study speculated that depression associated with high neuroticism scores might be a different subtype of depression than depression associated with vascular diseases and that late-life depression in the context of low neuroticism might be a marker of the latter but not the former [36].

In another study – examining the interaction between vascular disease and neuroticism as determinants of clinically relevant depressive symptoms in late-life – found that neuroticism was strongly associated with depressive symptoms in women but not in men, in which sex vascular disease attenuated the predictive power of neuroticism. The authors of this study speculated that the apathy caused by cerebrovascular disease might be responsible for the weakened association between neuroticism and depression in this population [37]. In the context of atherosclerosis, neuroticism, and late-life depression, the vascular apathy hypothesis has been confirmed [38].

To the best of our knowledge, this is the first study analyzing the role of Big Five personality traits and depression simultaneously in relation to PAD. The results indicated that neuroticism and depression were independently associated with PAD severity confirming that the relationship between PAD and depression cannot be explained by the influence of a general proneness to negative affectivity (neuroticism). Instead, the clinical presentation of depression has a distinct relationship with PAD, and vice versa: neuroticism as a stable trait has a distinct relationship with PAD independent of the specific symptoms of depression.

Depressive symptoms are associated with poorer adherence to lifestyle change recommendations suggested to individuals suffering from vascular diseases and consequently this mental disorder is also associated with a higher risk for and worse prognosis in PAD [39, 40]. However, depression is a modifiable risk factor: there are effective ways to reduce depression among patients with PAD [41]. Personality traits were shown to influence the process of recovery from depression: higher neuroticism predicted worse long-term recovery from depression [42] and was associated with lower remission rates in older patients treated for depression [43]. Some studies also suggested that neuroticism would also be a modifiable risk factor. The findings of Tang and colleagues showed that cognitive therapy as well as pharmacological antidepressant treatment can reduce neuroticism and increase extraversion in depressed patients [44]. The same authors also showed that internet-based cognitive therapy may result in long-term changes in personality traits related to neuroticism [45].

These results suggest that screening for depression and neuroticism in patients with PAD and offering treatment for these conditions could significantly improve patient outcomes and quality of life underlying the importance of an interdisciplinary model of care that integrates vascular medicine and mental health care for patients with PAD. Patients screened for mental health in an early phase of PAD could be provided with treatment which in turn may lead to fewer patients facing poor quality of life.

A major strength of the present study was the nuanced operationalization of PAD. Although the PAD subcategories employed in the present analyses are in general use in clinical practice, none of the previous studies considered the ABI-negative-symptomatic or the non-compressible-artery condition when investigating the relationship between depression and PAD, although individuals with these different presentations of PAD have the same mortality rate [46].

Limitations of the present study also need to be acknowledged. First, the cross-sectional design of the present study does not allow firm conclusions to be drawn regarding the direction of the relationship between PAD and depression or neuroticism. Further research with longitudinal designs could help us better understand the exact nature of the complex relationship between personality, depression, and vascular health. Secondly, the recruitment of participants was not systematic or random and occurred in the primary care setting; therefore, the findings of the present study cannot be generalized to the general or other specific populations. Further, the simultaneous presence of a relatively large number of independent variables and the relatively small sample of individuals in the ‘ABI-negative-symptomatic’ and the ‘Non-compressible-artery’ group also limits the reliability of the regression models for these subgroups. Finally, the post hoc power analyses indicated that our sample was not large enough to detect potentially extant, significant relationships between certain Big Five personality traits and peripheral arterial disease status. Adequately powered future studies need to confirm the findings of this explorative study.

## Conclusions

Despite these limitations, results of the present study call our attention to the fact that people with PAD – even those with asymptomatic forms of the disease – are at higher risk for depression compared to people without PAD, independently of neuroticism. These results underscore the importance of a multidisciplinary approach when providing care for individuals suffering from PAD to prevent poor prognosis either in the somatic or mental health domain.

## Data Availability

The dataset analyzed in the current study is not publicly available due to lack of consent from study participants to do so but it is available from the corresponding author on reasonable request for researchers who meet the criteria for access to confidential data.

## Abbreviations

PAD: Peripheral arterial disease
ABI: Ankle-brachial index CVD: Cardiovascular disease
CVD: Cardiovascular disease
